# Mitochondria‐Associated Endoplasmic Reticulum Membranes as Potential Therapeutic Targets in Epilepsy

**DOI:** 10.1002/cns.70726

**Published:** 2025-12-28

**Authors:** Huaiyu Sun, Xuewei Li, Weixuan Zhao, Wuqiong Zhang, Hongmei Meng

**Affiliations:** ^1^ Department of Neurology The First Hospital of Jilin University Changchun Jilin China; ^2^ Department of Radiology The First Hospital of Jilin University Changchun Jilin China

**Keywords:** endoplasmic reticulum, epilepsy, mitochondria, mitochondria‐associated endoplasmic reticulum membranes, therapeutic targets

## Abstract

**Background:**

Mitochondria‐associated endoplasmic reticulum membranes (MAMs) are specialized regions in cells where the endoplasmic reticulum and mitochondria closely interact. MAMs are enriched with a variety of proteins that regulate key cellular processes. These processes include mitochondrial fission and fusion, autophagy, lipid metabolism, calcium homeostasis, and oxidative stress. Increasing evidence suggests that disruption of MAMs structure and alterations in associated protein expression patterns are closely related to the pathogenesis of epilepsy.

**Methods:**

This review synthesizes and analyzes current literature to outline the structural and functional roles of key MAMs proteins. It further examines experimental and clinical evidence linking MAMs dysregulation to epileptogenesis and treatment responses.

**Results:**

The analysis confirms that MAMs serve as a central hub coordinating cellular homeostasis. Specific alterations in MAMs structure and protein expression are consistently associated with epilepsy models. These alterations directly impact neuronal excitability, synaptic function, and cell survival pathways involved in disease progression.

**Conclusion:**

Addressing these structural and functional properties of MAMs may provide valuable insights for developing novel therapeutic strategies for epilepsy.

## Graphical abstract

MAMs are involved in the occurrence and development of epilepsy through calcium ion homeostasis imbalance, mitochondrial dysfunction, endoplasmic reticulum stress and inflammation, abnormal lipid metabolism, as well as autophagy and mitochondrial autophagy. This picture was drawn using Figdraw.

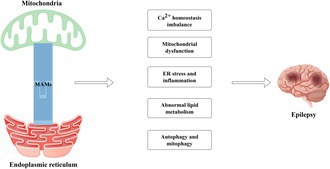

## 1 Introduction

Epilepsy is a common neurological disorder affecting approximately 70 million people worldwide [1]. Drug therapy, primarily involving various antiseizure medications, remains the mainstay of treatment. However, approximately one‐third of patients experience pharmacoresistant epilepsy, failing to achieve adequate seizure control with current medications [2, 3]. This highlights the urgent need to identify new therapeutic targets and develop innovative treatment strategies.

Recent research has increasingly focused on molecular mechanisms and potential targets that may lead to more effective epilepsy treatments. Non‐receptor tyrosine kinases have been implicated in epilepsy pathogenesis, suggesting that targeted small‐molecule inhibitors could offer promising therapeutic potential [4]. Additionally, the purinergic P2X7 receptor has emerged as a viable target, with animal studies indicating that its blockade potentially reduces seizure severity [5]. Moreover, phytotherapeutic approaches using medicinal plants and their extracts have demonstrated the ability to modulate pathways involved in epilepsy [6]. This multi‐target strategy addressing the complex and multifactorial nature of epilepsy may be particularly beneficial for patients with pharmacoresistant epilepsy.

Mitochondria‐associated endoplasmic reticulum membranes (MAMs) are specialized regions where the endoplasmic reticulum (ER) and mitochondria come into close contact, enabling the exchange of lipids and Ca^2+^ between these organelles [7]. This inter‐organelle communication is essential for sustaining cellular energy balance, redox homeostasis, and coordinating various physiological functions [8]. A deeper understanding of the roles and interactions between mitochondria and ER is crucial for elucidating disease mechanisms and developing therapeutic strategies targeting these organelles [9].

## 2 MAMs Structure and Function

MAMs are well‐characterized suborganelle contact sites that serve as critical interfaces between the ER and mitochondria. These specialized regions are not merely structural; they function as dynamic hubs that facilitate a variety of biological activities essential for maintaining cellular homeostasis. MAMs are enriched in a diverse number of proteins involved in key processes such as Ca^2+^ signaling, lipid metabolism, and mitochondrial dynamics (Table 1).

**TABLE 1:** MAMs‐related proteins and their functions.

MAMs proteins	Function
MFN1	Regulates mitochondrial fusion
MFN2	Regulates mitochondrial fusion
Drp1	Regulates mitochondrial division
IP3R	Regulates Ca^2+^ transport
Grp75	Regulates Ca^2+^ transport
VDAC	Regulates Ca^2+^ transport
Fundc1	Regulates Ca^2+^ transport
VAPB	Involved in Ca^2+^ transport, regulates autophagy, and affects synapses
PTPIP51	Involved in Ca^2+^ transport, regulates autophagy, and affects synapses
PERK	Participates in MAMs formation, regulates lipid and Ca^2+^ transport
α‐Syn	Maintains MAMs structure
PINK1	Involved in mitochondrial autophagy
Parkin	Involved in mitochondrial autophagy

Abbreviations: Drp1, dynamin‐related protein 1; Fundc1, FUN14 domain‐containing protein 1; Grp75, glucose‐regulated protein 75; IP3R, inositol 1,4,5‐trisphosphate receptor; MAMs, mitochondria‐associated endoplasmic reticulum membranes; MFN1, mitofusin 1; MFN2, mitofusin 2; Parkin, E3 ubiquitin‐protein ligase Parkin; PERK, protein kinase RNA‐like endoplasmic reticulum kinase; PINK1, PTEN‐induced kinase 1; PTPIP51, protein tyrosine phosphatase‐interacting protein 51; VAPB, vesicle‐associated membrane protein‐associated protein B; VDAC, voltage‐dependent anion channel; α‐Syn, alpha‐synuclein.

**FIGURE 1:**
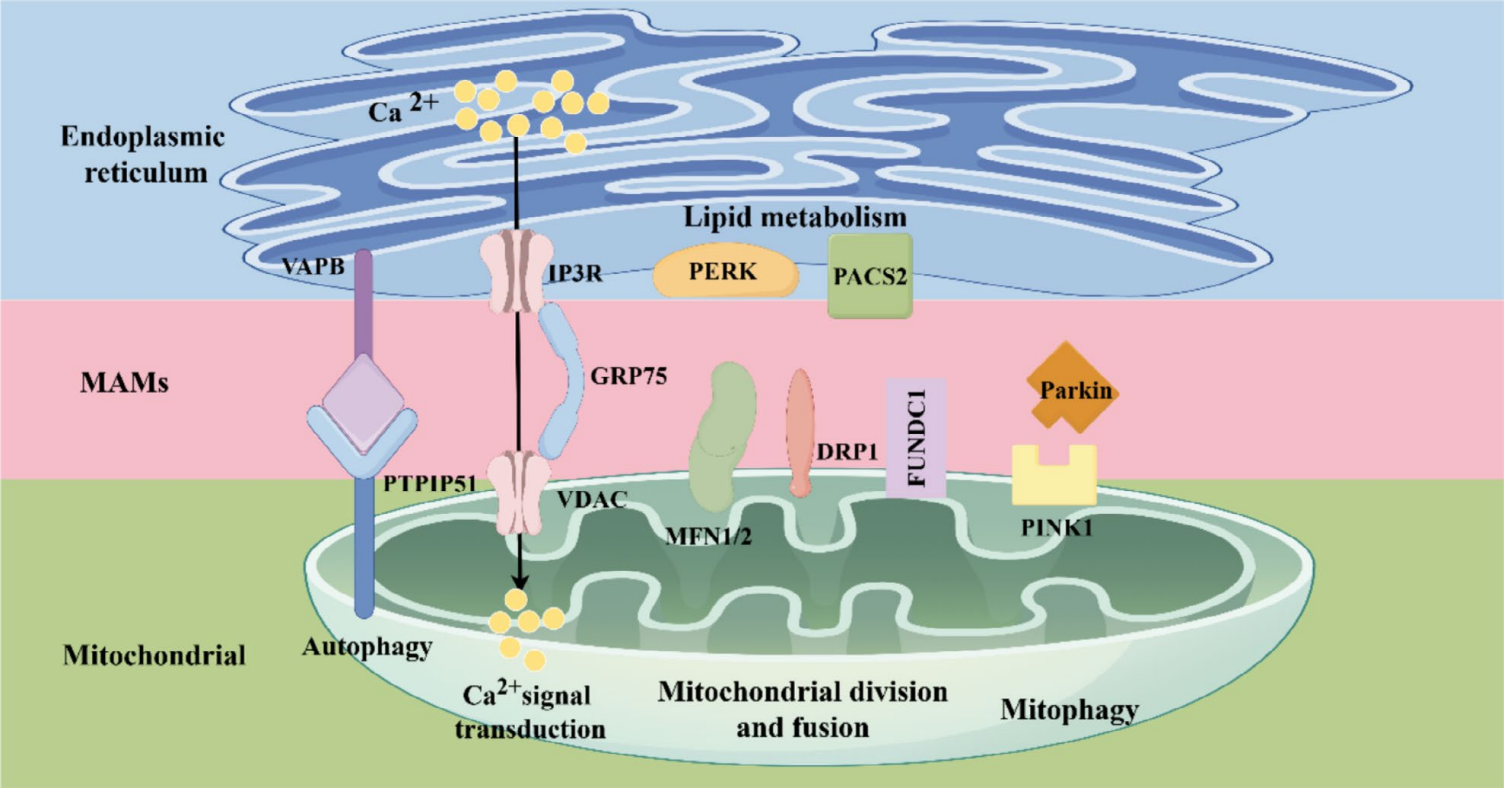
Primary tethering protein complexes responsible for maintaining mitochondria‐associated endoplasmic reticulum membranes (MAMs). MAMs are mainly involved in processes such as mitochondrial fusion and decomposition, lipid metabolism, Ca^2+^ transport, autophagy, and mitochondrial autophagy. This scheme is drawn using Figdraw.

Recent research has clarified the molecular composition of MAMs, identifying various tethering proteins that enable proximity to the ER and mitochondrial membranes. This proximity is crucial for effective inter‐organelle communication, enabling the transfer of lipids and ions necessary for cellular energy production and metabolic regulation [10, 11]. Briefly, MAMs are essential structures for organelle communication that impact numerous cellular functions and significantly contribute to the pathophysiology of various diseases. Understanding the mechanisms regulating MAMs dynamics and their related proteins may offer novel insights into therapeutic approaches for diseases associated with mitochondrial dysfunction (Figure 1).

### 2.1 Molecular Composition and Key Proteins of MAMs

#### 2.1.1 Ca^2+^ Channel Complex

Ca^2+^ is secreted from the ER to the mitochondria via the outer membrane and is subsequently transported into the mitochondrial matrix through unidirectional transporters in the inner membrane.

**FIGURE 2:**
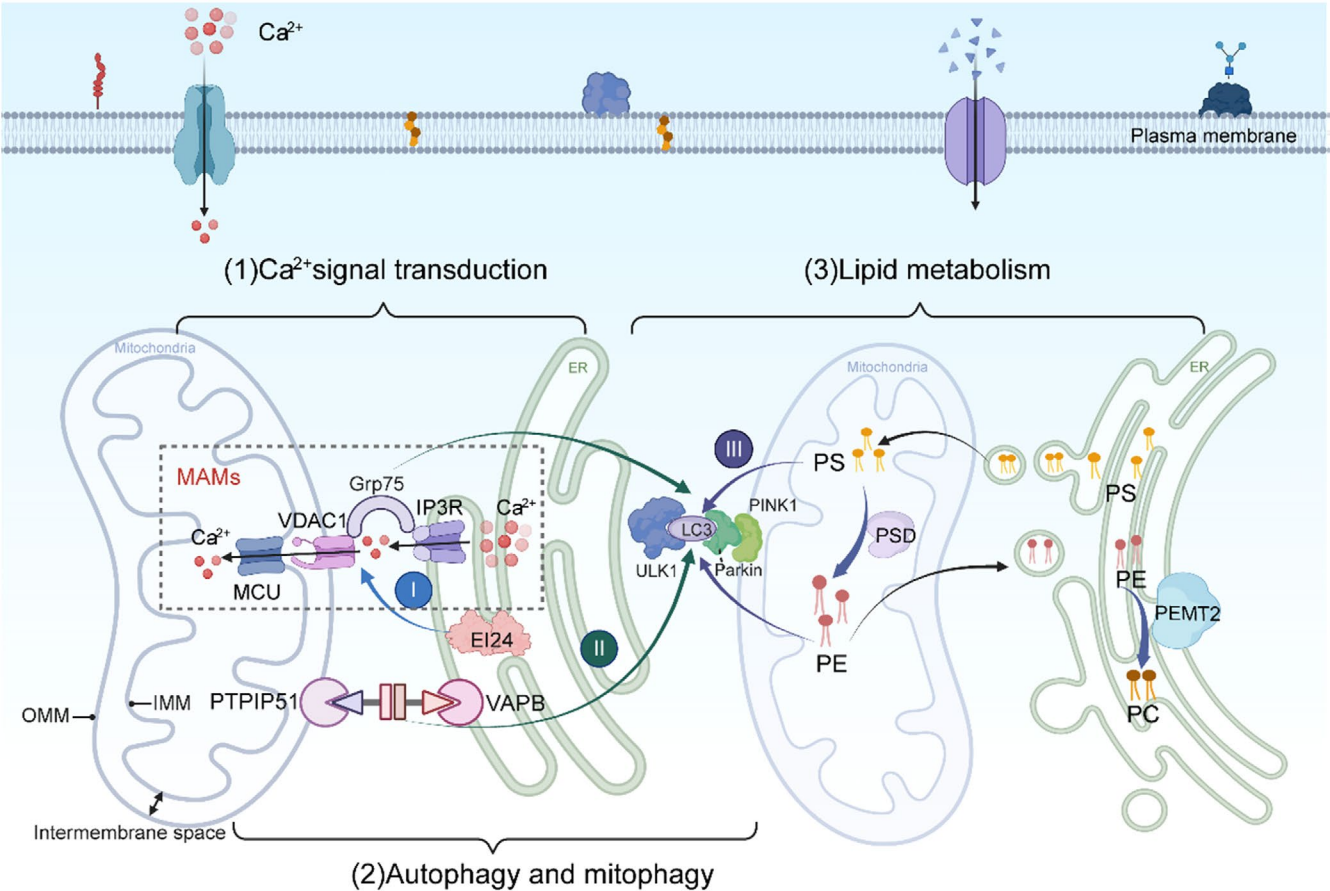
Ca^2+^ signal transduction, autophagy and mitophagy, and lipid metabolism at the mitochondria‐associated endoplasmic reticulum membranes (MAMs). OMM, outer mitochondrial membrane; IMM, inner mitochondrial membrane. Figure is created using BioRender. (1) Ca^2+^ is transferred from the ER to the mitochondria via the outer membrane and is subsequently transported into the mitochondrial matrix through unidirectional transporters in the inner membrane. IP3R at the MAMs is linked to VDAC1 at the OMM via grp75 in the cytosol. Meanwhile, high Ca^2+^ levels at the MAMs can promote Ca^2+^ uptake into the mitochondria by triggering the MCU on the IMM. (2) MAMs mediate autophagy and mitophagy. The ULK1 complex promotes the binding of Atg14 and Beclin1 to initiate autophagosome formation, and Atg5 is responsible for elongating the autophagosome membrane and promoting the translocation of LC3 across the membrane. Mitophagy dependent on Parkin/PINK1 is essential for initiating the removal of mitochondria. Located on the OMM, PINK1 draws in Parkin and increases its E3‐ubiquitin ligase activity, which leads to the ubiquitination of proteins on the OMM that mark damaged mitochondria. (I) EI24 interacts with the IP3R‐Grp75‐VDAC complex, affecting Ca transport and further influencing autophagy. (II) Overexpression or trial use of siRNA to influence VAPB or PTPIP51 levels can also affect autophagy. (III) Phosphatidylethanolamine (PE) is the main target of LC3. LC3 is linked to PE through its C‐terminal glycine residue. A high level of PE can promote the connection between PE and ATG8, facilitating the fusion and closure of phagocytic vesicles mediated by ATG8. (3) MAMs mediate lipid transport, synthesis, and metabolism. Phosphatidylserine (PS) is synthesized in MAMs and then transported from the endoplasmic reticulum to IMM. Under the action of PS decarboxylase in the inner mitochondrial membrane (IMM), the PS transferred to mitochondria is converted into PE. PE is then translocated back to the endoplasmic reticulum and eventually converted into phosphatidylcholine (PC) through PE‐N‐methyltransferase.

The inositol 1,4,5‐triphosphate receptor (IP3R), located on the ER membrane, regulates Ca^2+^ release into the cytoplasm and is crucial for cell survival and apoptosis. IP3R regulates directional Ca^2+^ transmission by interacting with the voltage‐dependent anion channel (VDAC) on the outer mitochondrial membrane (OMM) via glucose‐regulated protein 75 (GRP75). This mechanism influences the dynamic balance of intracellular Ca^2+^ and modulates cellular responses to a wide range of physiological and pathological stimuli (Figure 2). The function of IP3R is closely tied to the ER's Ca^2+^ storage capacity, with Ca^2+^ release being facilitated by IP3R activation, which is finely regulated by multiple signaling pathways [12, 13].

#### 2.1.2 Lipid Function‐Related Proteins

Key proteins in the ER and mitochondria are crucial for lipid synthesis and metabolism and significantly influence MAMs morphology and function. PC and PE are the primary structural lipids in biofilms. PS is synthesized in MAMs and is subsequently transported from the ER to IMM, where it is converted to PE [14]. Under the action of PS decarboxylase, a mitochondrially localized enzyme in the IMM, PS translocated to the mitochondria is converted into PE. PE then translocates back to the ER and is ultimately converted into PC through the enzyme PE‐N‐methyltransferase [15] (Figure 2).

The phosphofurin acidic cluster sorting (PACS) protein family, comprising PACS‐1 and PACS‐2, includes multifunctional proteins, with PACS‐2 specifically localized at the ER–mitochondria interface. PACS‐2 plays a crucial role in regulating MAMs, which are essential for cellular functions such as Ca^2+^ signaling, lipid metabolism, and autophagy [16]. Loss of PACS‐2 has been associated with MAMs disruption, potentially exacerbating renal tubular injury in diabetic nephropathy [17]. Moreover, PACS‐2 enhances mitochondrial function and supports cellular metabolic homeostasis by facilitating ER–mitochondria contact [18]. In diabetic models, PACS‐2 expression was positively associated with kidney function, indicating its crucial role in maintaining kidney health [17].

#### 2.1.3 MAMs Tethers

Mitofusin2 (MFN2), a mitochondrial GTPase, plays a crucial role in mitochondrial fusion required for sustaining mitochondrial function and cellular energy metabolism. Endoplasmic reticulum‐resident MFN2 forms homodimers or heterodimers with mitofusin1 or itself on OMM to promote mitochondrial fusion [19, 20]. In mammals, the association between endoplasmic reticulum mitochondria and their structures is more complex. Multiple protein complexes regulate the formation of MAMs by acting as endoplasmic reticulum‐mitochondrial junction complexes. These protein complexes include IP3R‐Grp75‐VDAC complex and vesicle‐associated membrane protein‐binding protein B (VAPB)‐tyrosine phosphatase‐interacting protein 51 (PTPIP51) complex [21, 22].

### 2.2 MAMs Detection Methods

With the advancement of MAMs research across various diseases, MAMs detection methods have also improved (Table 2). Because MAMs are localized in the contact regions between the ER and mitochondria, effective detection requires specialized methods to label these regions.

**TABLE 2:** MAMs detection methods and characteristics.

Detection method	Description	Advantages	Application areas
Co‐IP	Captures proteins associated with MAMs using specific antibodies and detects protein interactions using Western blot or mass spectrometry	Verifies protein complexes within MAMs and helps identify protein interactions	Protein–protein interactions, identification of MAMs‐related proteins
Fluorescence imaging	Uses fluorescently labeled antibodies to label MAMs‐related proteins and visualizes ER–mitochondria contact regions under fluorescent microscopy	Provides a direct visualization of MAMs locations and distributions	Cellular localization analysis, MAMs structural observations
EM	Utilizes electron microscopy, especially cryo‐EM, to observe the ultrastructure of ER–mitochondrial contact sites at high resolution	High resolution, enables direct observation of physical contact between ER and mitochondria	Ultrastructural observation, analysis of MAMs physical contact
Split‐GFP‐based contact site sensor	Uses split GFP to detect organelle interactions by reconstituting GFP fluorescence when organelles come into close contact	High sensitivity Real‐time, live‐cell imaging High spatial resolution	Studying MAMs (mitochondria–ER contact sites). Analyzing organelle interaction dynamics Investigating signaling, lipid metabolism, and calcium regulation
PLA	Detects protein–protein interactions using two primary antibodies, each linked to a unique oligonucleotide. When the target proteins are in proximity, the oligonucleotides ligate, generating a detectable signal	High sensitivity for detecting weak or transient interactions Enables detection in fixed or live cells Spatially resolved with high specificity	Studying protein–protein interactions Investigating signaling pathways Cellular localization of interactions Disease research (e.g., cancer, neurodegenerative diseases)
Calcium imaging	Uses calcium indicators to observe dynamic changes in intracellular calcium ion concentration, studying the calcium transfer between ER and mitochondria	Real‐time observation of calcium dynamics, indirectly assessing MAMs functional status	Calcium signaling, functional analysis of MAMs

Abbreviations: Co‐IP, co‐immunoprecipitation; EM, electron microscopy; ER, endoplasmic reticulum; GFP, green fluorescent protein; MAMs, mitochondria‐associated endoplasmic reticulum membranes; PLA, proximity ligation assay.

## 3 Potential Role of MAMs in Epilepsy

### 3.1 MAMs Role in Ca^2+^ Homeostasis Imbalance

Impaired Ca^2+^ homeostasis is a key factor in epilepsy pathogenesis, contributing to both neuronal hyperexcitability and damage following prolonged seizures. Ca^2+^ is vital for neuronal signaling, and abnormal fluctuation in its concentration can increase neuronal excitability, potentially leading to seizures [23]. Excessive Ca^2+^ influx activates multiple signaling pathways, disrupting the intracellular environment and triggering apoptosis or other forms of cell death [24]. Additionally, dysregulation of Ca^2+^ homeostasis can impair glial cell function, further compromising neuronal health and function, creating a self‐perpetuating cycle of dysfunction [25].

**FIGURE 3:**
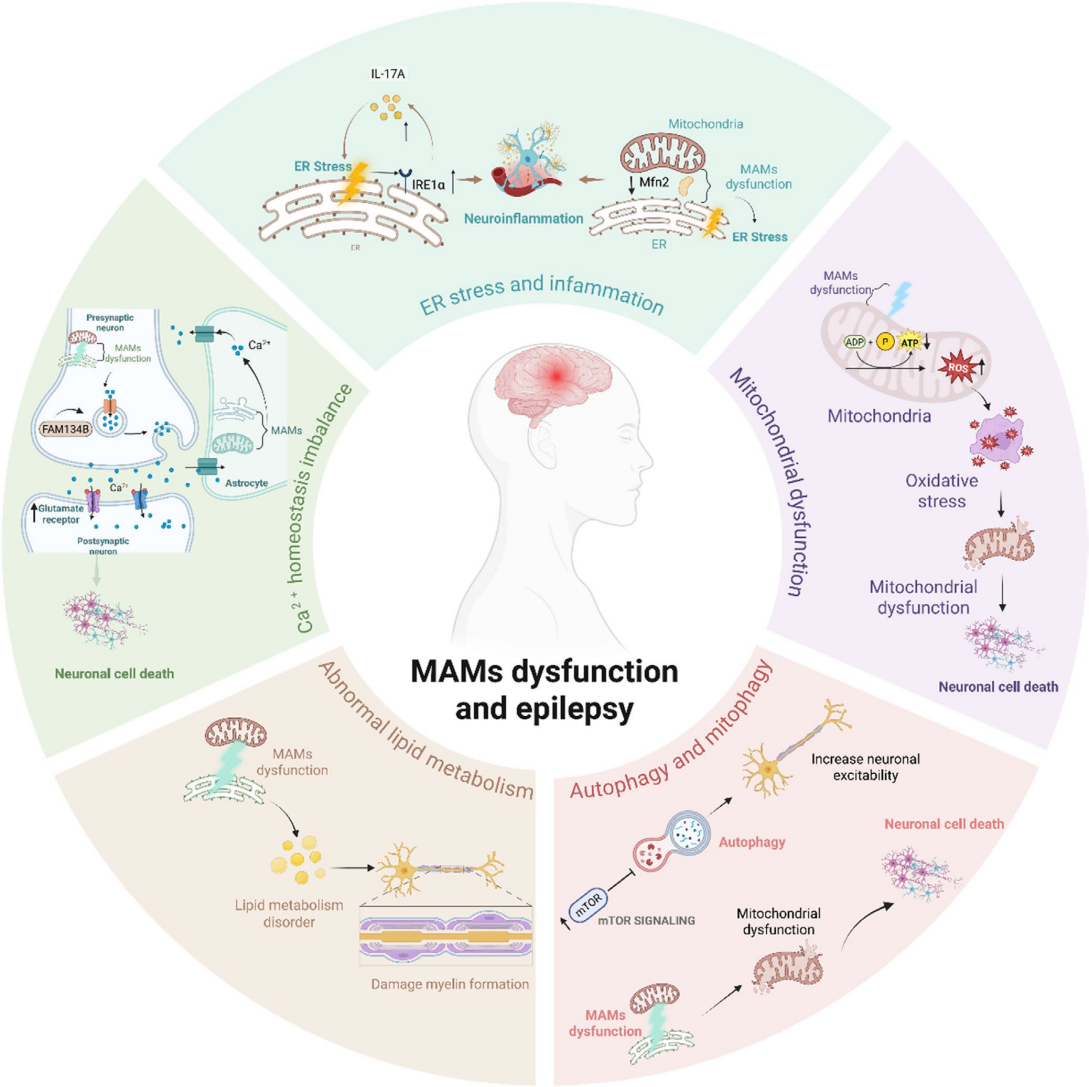
The role and mechanism of mitochondrial‐associated endoplasmic reticulum membranes (MAMs) in epilepsy. ER, Endoplasmic reticulum; MFN2, Mitofusin 2; ROS, Reactive oxygen species. Figure is created using BioRender.

MAMs dysfunction can disrupt Ca^2+^ signaling, leading to excitotoxicity characterized by excessive glutamate receptor activation and neuronal damage. Family sequence similarity 134, membrane B (FAM134B)‐mediated ER‐phagy plays a crucial role in balancing mitochondrial Ca^2+^ levels and regulating cell death in epileptic hippocampal neurons (Figure 3). Therefore, boosting ER‐phagy may be a therapeutic strategy for preventing neuronal apoptosis in epilepsy [26].

Disrupted Ca^2+^ homeostasis is also associated with the activation of signaling pathways that lead to neuronal hyperexcitability. IP3R facilitates Ca^2+^ signaling between the ER and mitochondria. Disruption of this signaling axis can lead to mitochondrial dysfunction and increased neuronal excitability, further exacerbating seizure risk [27]. Excessive Ca^2+^ influx during excitotoxicity can surpass cellular defenses, resulting in neuronal death and epilepsy.

Furthermore, astrocytes regulate neuronal Ca^2+^ dynamics through their interactions with MAMs. Astrocytes play a critical role in maintaining extracellular ion balance and modulating synaptic activity. Abnormal astrocyte feedback can lead to pathological changes in neuronal firing patterns, contributing to seizure onset [28]. Therefore, glial cells are important in epilepsy, and their dysfunction can exacerbate the effects of MAMs‐related Ca^2+^ dysregulation.

### 3.2 Mitochondrial Dysfunction

Mitochondrial dysfunction plays a critical role in epilepsy pathogenesis, affecting energy metabolism, oxidative stress, Ca^2+^ homeostasis, and genetic mutations. Neurons rely heavily on mitochondrial energy production, and impaired mitochondrial function can lead to energy deficits that disrupt neuronal activity and survival [29, 30]. Additionally, mitochondria help regulate intracellular Ca^2+^ levels, and disturbances in Ca^2+^ homeostasis are key contributors to seizures [31]. Oxidative stress, often driven by excessive ROS secreted from dysfunctional mitochondria, can further damage cells and promote neuronal death.

Disruption of ER–mitochondria interaction may result in mitochondrial dysfunction, marked by impaired oxidative phosphorylation and increased ROS production [32]. Oxidative stress can exacerbate neuronal damage, potentially contributing to epilepsy development by enhancing excitotoxicity and neuroinflammation. In epilepsy, mitochondrial dysfunction has been linked to energy metabolism disorders that affect neuronal survival and function. Changes in mitochondrial bioenergetics can lead to decreased ATP production, which is critical for maintaining neuronal excitability and synaptic transmission [33, 34]. Impaired mitophagy results in the build‐up of damaged mitochondria, which can activate apoptotic pathways, causing neuronal cell death and heightened susceptibility.

Recent research has demonstrated the therapeutic potential of interventions targeting mitochondrial function and MAMs integrity in epilepsy. Antioxidants that reduce oxidative stress and enhance mitochondrial function are promising neuroprotective strategies. Additionally, compounds that modulate MAMs dynamics and promote mitochondrial biogenesis can help restore energy metabolism in neurons, thereby mitigating seizure risk [35].

Elucidating the interaction between MAMs, mitochondrial damage, and neuronal energy metabolism is crucial for understanding the pathophysiology of epilepsy. Targeted therapies addressing metabolic disturbances could offer novel strategies for epilepsy prevention and treatment, emphasizing the crucial role of mitochondrial health in sustaining neuronal function and preventing excitotoxicity.

### 3.3 ER Stress and Inflammation

ER stress and neuroinflammation significantly contribute to epilepsy pathogenesis. Together, they disrupt neuronal function, destabilize neural networks, and impact glial responses. Neuroinflammation can cause increased neuronal excitation and exacerbate epileptic pathology by altering glial cell function [36]. During seizures, glial cells release increased levels of proinflammatory cytokines, which further enhance neuronal excitability and create a self‐perpetuating cycle of inflammation and seizure activity [37]. ER stress is also a key trigger of neuroinflammation, which, in turn, contributes to epilepsy progression [38, 39]. Understanding the complex interplay between ER stress and neuroinflammation is crucial for developing new strategies for the treatment of epilepsy [40, 41].

Recent research has underscored the link between MAMs dysfunction, ER stress, and heightened inflammatory responses, which can significantly impact the pathophysiology of epilepsy. Characterizing the interactions among these cellular mechanisms is crucial for understanding how ER stress contributes to heightened seizure susceptibility and epilepsy progression.

ER stress activates signaling pathways that promote inflammation. Activation of the inositol‐requiring enzyme 1 alpha (IRE1α) pathway during ER stress can increase proinflammatory cytokine production, potentially worsening neuroinflammation and neuronal damage in epilepsy [42]. Interleukin (IL)‐17A enhances ER stress‐mediated inflammation in macrophages, indicating that inflammatory cytokines may intensify stress responses in neuronal tissues [43]. This inflammatory milieu can create a vicious cycle, in which inflammation leads to increased ER stress, which in turn promotes further inflammatory responses.

MAMs dysfunction is linked to the development of neurological disorders, including epilepsy. MFN2, a protein that maintains MAMs integrity, regulates mitochondrial function and ER stress responses. Its downregulation in certain conditions—such as diabetic kidney disease—has been associated with increased apoptosis and ER stress, indicating the importance of MAMs integrity in cellular resilience against stressors [44]. In epilepsy, the loss of MAMs integrity may lead to increased neuronal vulnerability to excitotoxicity and inflammation, thereby increasing seizure susceptibility.

The relationship between peripheral inflammation and seizure susceptibility has also been explored. Peripheral inflammation can trigger neuroinflammation and oxidative stress in the hippocampus, a key area involved in seizure generation. Therefore, systemic inflammatory responses may have localized effects on brain function, potentially leading to increased seizure frequency and severity [45, 46]. Modulating inflammatory pathways, including tumor necrosis factor‐alpha and IL‐1β, can impact seizure outcomes, underscoring the significance of targeting inflammation as a therapeutic approach in the management of epilepsy [47].

### 3.4 Abnormal Lipid Metabolism

MAMs are vital for lipid transport and membrane composition regulation, both of which are crucial for cellular homeostasis. Lipids are not only structural components of membranes but also serve as signaling molecules in neuronal excitability. For instance, membrane lipid composition can affect the function of ion channels, including those involved in generating action potentials. Changes in lipid profiles at MAMs may lead to dysregulation of this activity, which is a hallmark of epileptic seizures [48].

Moreover, the role of MAMs in lipid transport is critical for the synthesis of myelin and the protective sheath around axons. Myelin integrity is essential for proper neuronal signaling, and disturbances in lipid metabolism can impair myelination, potentially leading to neurological disorders, including epilepsy. Specific lipid classes, such as sphingolipids and phospholipids, are crucial for maintaining myelin structure and function [49].

Furthermore, the interaction between MAMs and other cellular organelles, such as lipid droplets and the Golgi apparatus, demonstrates the complexity of lipid metabolism in neurons. These interactions facilitate the efficient transport and distribution of lipids required for membrane biogenesis and repair. Disruptions in these pathways can exacerbate the pathological conditions associated with epilepsy. Therefore, MAMs are important for maintaining lipid homeostasis [50].

### 3.5 Role of MAMs in Autophagy and Mitophagy

MAMs dysfunction can disrupt autophagic flux, potentially worsening neuronal damage during epileptic episodes. Exposure to toxic agents can disrupt MAMs integrity, leading to mitochondrial dysfunction and subsequent neuronal apoptosis, which is often accompanied by increased levels of autophagy‐related markers; thus, while autophagy may initially act as a protective mechanism, its dysregulation can contribute to neuronal death in epilepsy [51, 52].

The mechanistic target of rapamycin (mTOR) pathway, a crucial regulator of autophagy, is intimately associated with MAMs function [53, 54]. Excessive mTOR activation is linked to epilepsy development by suppressing autophagy and increasing neuronal excitability. Inhibition of mTOR signaling enhances autophagic activity, potentially offering neuroprotective effects against seizures [52, 55]. Therefore, therapeutic strategies aimed at modulating MAMs function and autophagy may be beneficial for the management of epilepsy.

The interaction between MAMs and autophagy is further complicated by various signaling pathways, including those associated with oxidative stress and inflammation. For example, MAMs are implicated in the regulation of Ca^2+^ signaling, which is crucial for autophagy initiation. Dysregulation of Ca^2+^ homeostasis can lead to impaired autophagy and increased neuronal vulnerability during seizures [56, 57].

## 4 Potential Therapeutic Targets for MAMs

### 4.1 Ca^2+^ Ion Complex

Small molecule drugs targeting VDAC are essential for regulating intracellular Ca^2+^ flow and mitochondrial function. In vitro and in vivo studies have identified a group of VDAC antagonists (VA molecules) that selectively bind to VDAC1, demonstrating specificity for cancer cells. VA molecules bind to VDAC1, disrupting the NADH binding site, thereby resulting in mitochondrial dysfunction and decreased cell proliferation compared with that in non‐cancer cells [58]. In epilepsy, dysregulation of Ca^2+^ homeostasis can lead to neuronal hyperexcitability and seizures. The interaction between VDAC and other proteins, such as the antiapoptotic protein Bcl‐xL, enhances mitochondrial Ca^2+^ uptake, thereby influencing neuronal survival and function during epileptic events. Studies have shown that modulating IP3R activity can influence Ca^2+^ release and potentially serve as a therapeutic target for epilepsy. For instance, targeting IP3R‐mediated Ca^2+^ signaling pathways might help in reducing seizure frequency and severity by stabilizing neuronal Ca^2+^ homeostasis and preventing excessive neuronal firing [59].

### 4.2 Mitochondrial Kinetic Regulation

Targeting MFN2 has demonstrated potential in the context of mitochondrial disease‐related epilepsy. The investigational small‐molecule therapeutic EPI‐743 decreased seizure frequency in mitochondrial disease‐related epilepsy, suggesting a potential treatment strategy through MFN2 regulation [60]. The epigenetic regulation of MFN2, including DNA methylation, has been studied in relation to mitochondrial dysfunction caused by brain trauma, indicating that hypermethylation of the MFN2 gene promoter can downregulate its decreased leading to subsequent mitochondrial dysfunction. Therefore, demethylating agents could restore MFN2 function and improve cognitive deficits, providing a potential therapeutic avenue for epilepsy treatment [61].

In a rat model of pilocarpine‐induced status epilepticus, mitochondrial division inhibitor 1 (mdivi‐1), a selective small‐molecule inhibitor of dynamin‐related protein 1 (Drp1), significantly attenuated neuronal death in the hippocampus, suggesting that the inhibition of Drp1 can protect against cell death associated with seizures, possibly by inhibiting cytochrome c release, preventing apoptosis‐inducing factor translocation, and suppressing the mitochondrial apoptosis pathway [62]. Drp1 is also crucial in managing mitochondrial quality and cellular functions, particularly in the context of hypoxia/ischemia‐induced mitochondrial imbalance and injury, which shares pathophysiological features with epilepsy. Targeting Drp1 could provide new therapeutic strategies for mitigating mitochondrial dysfunction and neuronal damage in epilepsy.

Therefore, targeting Drp1 and MFN2 may be novel therapeutic strategies for epilepsy management, offering insights into how modulating mitochondrial dynamics can influence neuronal survival and function during epileptic events.

### 4.3 ER Stress

Targeting the PERK and IRE1 pathways offers a promising approach for treating ER stress‐related conditions, such as epilepsy. For instance, selective inhibition of PERK protects against ER stress‐induced apoptosis in diabetic cardiomyopathy, suggesting its potential to reduce neuronal cell death in epilepsy [63]. Similarly, targeting the IRE1 pathway mitigates ER stress and improves cell survival in various models, including those related to metabolic disorders [64]. Interactions between the PERK and IRE1 pathways can also impact outcomes of ER stress response. IRE1 signaling can maintain PERK expression during prolonged ER stress, illustrating the interconnectedness of these pathways and their combined influence on cell fate [65]. This interplay suggests that a combined therapeutic approach targeting PERK and IRE1 could be more effective in managing ER stress‐related conditions such as epilepsy.

The ketogenic diet (KD) is a high‐fat, low‐carbohydrate regimen that has garnered significant attention in recent studies on neurological disorders. First, in a study using a temporal lobe epilepsy (TLE) rat model, KD significantly reduced spontaneous recurrent seizures while improving learning and memory abilities. The findings indicate that KD suppresses ERS overactivity induced by epilepsy, protects neuronal endoplasmic reticulum ultrastructure, optimizes synaptic ultrastructure, and increases dendritic spine density in the hippocampus [66]. Secondly, the role of the ketogenic diet in endoplasmic reticulum stress and mitochondrial metabolism has also been validated. Studies have shown that the ketogenic diet can enhance cerebral ischemia tolerance by inhibiting Drp1‐mediated mitochondrial fission and endoplasmic reticulum stress while suppressing the activation of the NLRP3 inflammasome. In a mouse model of midbrain artery occlusion/reperfusion injury, the ketogenic diet significantly reduced endoplasmic reticulum stress and the activation of the TXNIP/NLRP3 inflammasome, thereby exerting neuroprotective effects [67].

### 4.4 Regulation of Lipid Metabolism

Phosphofurin acidic cluster sorting protein 2 (PACS‐2) is essential for lipid metabolism, and its deficiency can lead to lipid‐related neurological damage. PACS‐2 dysfunction may worsen epilepsy because of its significant role in lipid metabolism and neuronal activity [68]. Targeting PACS‐2 in epilepsy is an emerging research area. In addition, genes related to lipid metabolism in PACS‐2 are differentially expressed in patients with epilepsy, suggesting the role of PACS‐2 in epilepsy onset and progression by regulating lipid metabolic pathways [69]. Therefore, targeting the lipid metabolism of PACS‐2 may not only improve understanding of the pathophysiological mechanisms of epilepsy but also potentially provide new strategies and directions for its treatment.

Cholesterol 24‐hydroxylase is a cytochrome P450 (CYP46A1) that is selectively expressed in the brain and is responsible for the renewal of most cholesterol in the central nervous system. 24‐hydroxylase is located in the endoplasmic reticulum and is distributed in the cell bodies and dendrites of various neurons. Research has found that mice lacking 24‐hydroxylase exhibit learning disabilities and long‐term dysfunction of the hippocampus, indicating that the enzyme's metabolism of cholesterol is crucial for learning and memory formation [70]. During treatment with Soticlestat (a novel cholesterol 24‐hydroxylase inhibitor), the number of epileptic seizures was approximately three times less than that of mice treated with vehicles [71]. The pharmacological mechanism by which Soticlestat inhibits CYP46A1 may involve multiple molecular pathways. For instance, one of the multiple mechanisms associated with the occurrence of epilepsy is TrkB signaling [72], which involves CYP46A1 [73]. Another interesting example is the positive allosteric regulation of the NMDA receptor by 24S‐hydroxycholesterol [74].

### 4.5 Autophagy and Mitophagy as a Target of MAMs

The vesicle‐associated membrane protein‐associated protein B‐protein tyrosine phosphatase interacting protein 51 (VAPB‐PTPIP51) tethering complex is vital for ER–mitochondria interactions and is crucial for cellular homeostasis and the regulation of processes such as Ca^2+^ signaling and autophagy. Disruption of this complex is linked to neurodegenerative diseases, such as frontotemporal dementia and amyotrophic lateral sclerosis, through Ca^2+^ homeostasis and synaptic function [75, 76].

Modulating ER–mitochondria tethering via the VAPB‐PTPIP51 complex presents a potential new therapeutic strategy for epilepsy. This complex plays a role in Ca^2+^ signaling, which is relevant in epilepsy because Ca^2+^ dysregulation contributes to epileptic activity. Thus, enhancing the VAPB‐PTPIP51 interaction could potentially stabilize Ca^2+^ signaling and reduce neuronal excitability, thereby mitigating seizures [76].

Additionally, modulation of the VAPB‐PTPIP51 complex could impact autophagy, which is increasingly recognized for its neuroprotective role and effects on neuronal health. Autophagy dysfunction has been linked to epilepsy; hence, interventions that restore autophagic flux could provide therapeutic benefits. Modulating VAPB‐PTPIP51 tethering may enhance autophagic processes, thereby reducing the pathological accumulation of proteins and damaged organelles that contribute to epilepsy pathogenesis [77].

Overall, the VAPB‐PTPIP51 complex represents a promising target for therapeutic intervention in epilepsy, offering potential benefits by modulating Ca^2+^ signaling and autophagy. Further study of this complex may reveal new treatment ideas for targeting cellular dysfunctions linked to epilepsy.

## 5 Prospects

The therapeutic targeting of MAMs holds significant promise for the future of epilepsy treatment. Ongoing research may lead to more effective therapies, with advances in genomics and personalized medicine potentially identifying genetic mutations or variations impacting MAMs' function in epilepsy. Tailored therapies based on genetic and molecular profiling could maximize the efficacy and safety of MAMs‐targeted treatments. High‐throughput screening could be applied to identify small molecules or biologics that specifically modulate MAMs‐associated proteins, which may provide novel therapeutic options for patients with epilepsy, especially those who are resistant to traditional therapies. Combining MAMs‐targeted therapies with existing antiepileptic drugs or other therapeutic modalities, such as neurostimulation or gene therapy, could provide a synergistic effect that may enhance treatment outcomes and help reduce the need for polypharmacy, which often leads to adverse side effects.

## 6 Conclusions

MAMs play critical roles in cellular Ca^2+^ homeostasis, lipid metabolism, oxidative stress, energy metabolism, and apoptosis. These functions are intrinsically linked to the pathophysiological mechanisms underlying epilepsy. Research on MAMs holds the potential for transformative advances in epilepsy treatment. While MAMs dysfunction may increase neuronal excitability, modulating Ca^2+^ signaling, such as using IP3R targeting, can mitigate seizure frequency and severity. Drugs targeting MAMs, such as those stabilizing the VDAC‐IP3R‐GRP75 complex to reduce abnormal Ca^2+^ transfer, may offer greater precision than traditional Ca^2+^ channel blockers.

Epileptic seizures frequently result in mitochondrial dysfunction and increased ROS, causing cellular damage and inflammation. MAMs link the ER and mitochondria, acting as a key center for managing oxidative stress responses. MAMs regulate antioxidant proteins (e.g., superoxide dismutase 2) and redox pathways (e.g., the nuclear factor erythroid 2–related factor 2 [Nrf2] pathway). Targeting antioxidant pathways in MAMs (e.g., activating the Nrf2 pathway) can reduce seizure‐induced neuronal damage by regulating oxidative stress‐related proteins in MAMs (e.g., C/EBP homologous protein), thereby mitigating inflammation and apoptosis in neurons.

Patients with epilepsy often experience energy metabolism disorders, particularly insufficient ATP production and metabolic abnormalities in the brain, which are crucial for energy metabolism, thus influencing mitochondrial function, ATP synthesis, and metabolite transport, including adenine nucleotide translocator (ANT) and pyruvate dehydrogenase kinase 4. Developing drugs targeting MAMs metabolic proteins (e.g., ANT or steroidogenic acute regulatory protein 3 [StARD3]) may improve neuronal energy supply. Dietary interventions such as the ketogenic diet, which boosts mitochondrial metabolism, may influence MAMs functions, providing a more targeted therapeutic approach.

Abnormalities in lipid metabolism are associated with neuroinflammation and disrupted cellular signaling, potentially exacerbating epilepsy. MAMs serve as metabolic hubs for cholesterol, phospholipids, and sphingolipids, thereby regulating neuronal membrane stability and function. Targeting lipid transport proteins such as StARD3 or VAPB may reduce neuroinflammation caused by lipid metabolism disorders. Adjusting MAMs' function to optimize lipid metabolism may improve the stability of neuronal signaling.

Recurrent seizures induce excessive neuronal apoptosis and damage. MAMs are crucial for integrating apoptotic signals and controlling mitochondrial outer membrane permeability and proteins related to apoptosis, such as Bcl‐2, Bax, and Bcl‐2 antagonist/killer. Targeting Bcl‐2 family proteins within MAMs to develop novel anti‐apoptotic drugs can inhibit MAMs‐mediated apoptosis signals, thereby promoting neuronal survival.

The heterogeneity of the pathophysiological processes in epilepsy limits the effectiveness of traditional treatments such as antiepileptic drugs. MAMs' function can vary among patients depending on genetic and metabolic factors. MAMs‐related proteins (e.g., VAPB and VDAC1) may serve as early diagnostic biomarkers for epilepsy. Precision medical strategies tailored to a patient's MAMs functional status could significantly improve therapeutic outcomes.

In conclusion, MAMs provide a vital interface between the ER and mitochondria, playing essential roles in Ca^2+^ signaling, lipid metabolism, and apoptosis regulation. Disruption of MAMs function is increasingly linked to epilepsy pathogenesis, highlighting their potential as novel therapeutic targets. By modulating Ca^2+^ homeostasis, enhancing mitochondrial function, restoring lipid metabolism, and preventing neuronal autophagy, MAMs‐targeted therapies may provide a novel approach to treating epilepsy. However, further research is necessary to elucidate the precise mechanisms of MAMs dysfunction in epilepsy and develop safe and effective therapies. Incorporating MAMs‐targeted strategies into clinical practice could significantly advance treatment options, particularly for patients with drug‐resistant epilepsy.

## Author Contributions

H.S.: conceptualization, writing – original draft; X.L. and W.Z.: formal analysis, investigation, funding acquisition; W.Z. and H.M.: writing – reviewing and editing.

## Funding

This work was supported by the Department of Science and Technology of Jilin Province, 20240304165SF; Education Department of Jilin Province, 2025KC102.

## Conflicts of Interest

The authors declare no conflicts of interest.

## Data Availability

The data that support the findings of this study are available from the corresponding author upon reasonable request.
